# Structural characteristic of polysaccharide isolated from *Nostoc commune*, and their potential as radical scavenging and antidiabetic activities

**DOI:** 10.1038/s41598-022-26802-x

**Published:** 2022-12-22

**Authors:** Xinjian Wang, Zhen Yang, Yu Liu, Xuehong Wang, Hongjuan Zhang, Ruofeng Shang, Cidan Laba, Cuomu Wujin, Baocheng Hao, Shengyi Wang

**Affiliations:** 1 Key Laboratory of New Animal Drug Project, Gansu Province, Key Laboratory of Veterinary Pharmaceutical Development, Ministry of Agriculture and Rural Affairs, Lanzhou Institute of Husbandry and Pharmaceutical Sciences of Chinese Academy of Agriculture Sciences, Lanzhou, 730050 People’s Republic of China; 2 Institute of Animal Science and Veterinary, Tibet Academy of Agricultural and Animal Husbandry Sciences, Lhasa, 850009 People’s Republic of China

**Keywords:** Biochemistry, Astronomy and planetary science, Chemistry, Materials science, Physics

## Abstract

In this paper, *Nostoc commune* crude polysaccharide was extracted by heating and Ultrasonic-assisted methods separately, homogeneous polysaccharide HNCP3 and UNCP4 were obtained after purified by DEAE-52 cellulose column chromatography and Sephacryl G-100 gel column chromatography. The structures of HNCP3 and UNCP4 were characterized by molecular weight determination, infrared spectroscopy, DSC detection, sodium periodate oxidation, smith degradation reaction and methylation analysis. The conformation of the solution was studied by SEM and AFM. The results showed that the Ultrasonic-assisted extraction had effects on the molecular weight, monosaccharide composition, molar ratio and configuration of *Nostoc commune*. The main chain of HNCP3 and UNCP4 was → 6)-D-Glcp(1→ and → 2, 6)-D-Glcp, but UNCP4 contained 1, 2, 6-galactose and 2, 3-Me2-D-Ara branches, while HNCP3 did not. The results of the monosaccharides composition of indicated that mannose was presented in both HNCP3 and UNCP4. SEM and AFM showed that the structure of UNCP4 was helical, and the solution conformations of HNCP3 and UNCP4 were different in different solution environments. Studies on DPPH radicals, superoxide anions, and hydroxyl radicals scavenging abilities showed that UNCP4 had higher antioxidant activity, while studies on the antidiabetic activities showed that the hypoglycemic effect of UNCP4 was stronger than that of HNCP3. Therefore, Ultrasonic-assisted extraction (UAE) increases the bioactivity of *Nostoc commune* polysaccharide (NCP) as well as the extraction rate.

## Introduction

*Nostoc commune* is a species of lower heterocystous blue-green alga capable of forming an extended jelly layer consisted of polysaccharides, which grows vigorously in dry and cold barren mountainous regions^1,2^. *Nostoc commune* is rich in protein, polysaccharide, amino acids, lipids and a variety of vitamins and metal elements, with rich nutritional value. Many reports have proved that different species of *Nostoc* contain a variety of unique medicinal compounds (amino acids, fatty acids, polysaccharides, antimicrobial substances) that exhibit good bioactivities, such as nitrogen-fixing^3^, reducing the risk of coronary heart^4^, antioxidation^5^, anti-microbico^6^, suppressing the human T-lymphoid Jurkat cell growth^7^, anti-tumor^8^, etc. Until now, *N. commune* also has been revealed to be rich in proteins, sugars, and lipids, as well as trace elements and vitamins^9^, and has beneficial physiological functions such as anti-infection^10^, decreasing blood lipid levels^11^, activating autophagy by down regulating PI3K/AKT/mTOR pathway^12^, antimicrobial and anti-inflammatory^13^, etc. Hence, *N. commune*, commonly referred to as ‘‘ishikurage’’, has previously been used as food ingredient or folk medicine for prevention and cure illnesses in Southeast Asia^14^.

It has been pointed out that polysaccharides of *N. commune* play an important role in resisting extreme desiccation, readily restoring metabolic activity and having novel bioactivities upon rehydration*,* and these functions exhibit structure–activity relationship^15–20^. It is well known that the extraction method has a significant effect on the yield, structural characteristics and biological activities of polysaccharides^21,22^. The conventional heating reflux extraction (HRE) is the most commonly used method for extracting polysaccharides. Generally, the yield of this process is highly dependent on the extraction time and temperature^15,23^. However, the higher extraction temperature and longer extraction time may result in the degradation of polysaccharides and reduction of pharmacological activities^24^. Therefore, several new techniques have been employed to extract polysaccharides for increasing extraction rate^25^, such as ultrasonic-assisted extraction (UAE)^26–28^ and microwave-assisted extraction (MAE). However, there is few research has been conducted on the comparation of physical properties, structural features and bioactivities between the polysaccharides obtained by HRE and UAE. The ultrasound-assisted extraction technology has been used to obtain the crude polysaccharides from *Nostoc commune* in previous study^20^. In this study, the conventional heated reflux extraction, ultrasonic-assisted extraction and alcohol precipitation technology were used to prepare the crude polysaccharides (NCP) of *N. commune* which collected from cold-humid area at an altitude of about 2000 m (Weiyuan County, Gansu Province, China), and then the physicochemical properties, structural features and bioactivities of the purified segments purified by the chromatography were studied for evaluating the structure–activity relationships.

## Results and discussion

### Purification of HNCP3 and UNCP4 by chromatography technique

**Figure 1:**
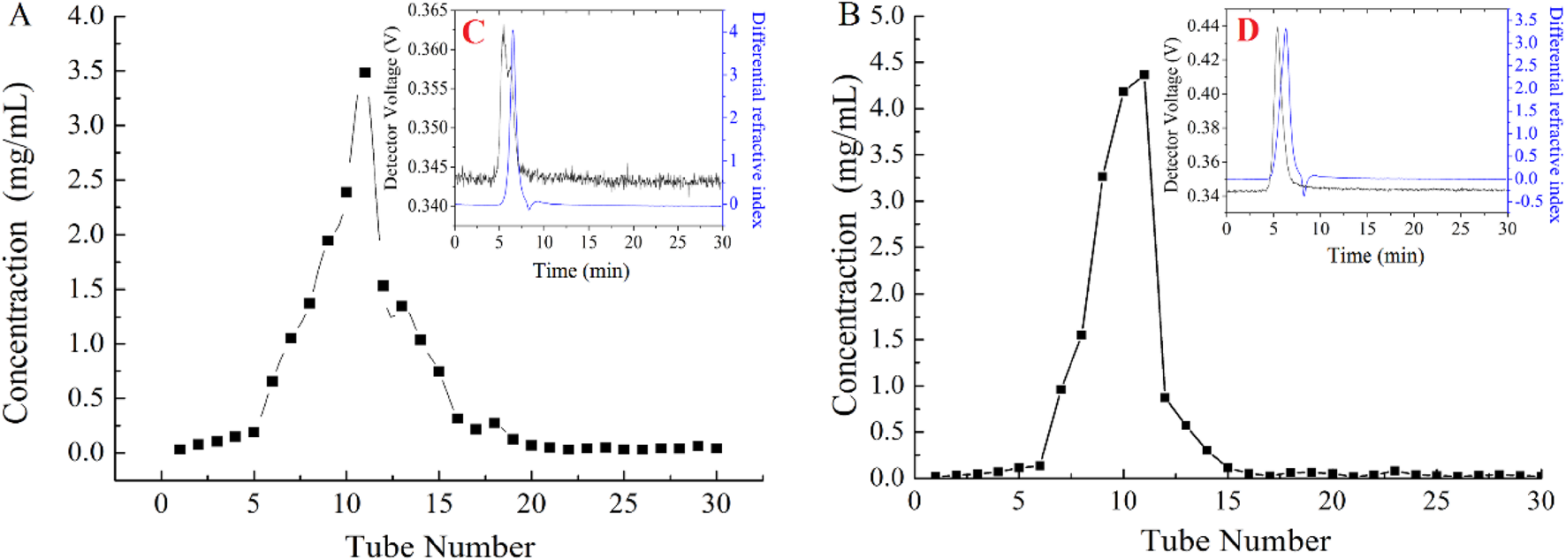
Elution profiles and molecular weight distributions of HNCP3 and UNCP4 purified by DEAE-52 cellulose column and Sephadex G-100 column. (**A**): The elution curve of HNCP3 purified by Sephadex G100; (**B**): The elution curve of UNCP4 purified by Sephadex G100; (**C**): Molecular weight distributions of HNCP3 by high-performance liquid chromatography coupled with multi-angle laser light scattering determination; (**D**): Molecular weight distributions of UNCP4 by high-performance liquid chromatography coupled with multi-angle laser light scattering determination.

10 mg/mL of HNCP3 and UNCP4 were loaded into the DEAE-52 chromatographic column respectively, and eluted with NaCl solution in the range of 0–1.0 mol/L. The elution with 0.3 mol/L of NaCl was collected and traced by the phenol sulfuric acid method, two peaks were obtained with the recoveries of 29.34% and 34.28%. After dialysis and lyophilization, the sample was further purified by Sephadex G 100 chromatography column, two single narrow symmetrical peaks named HNCP3 and UNCP4 were obtained (Fig. 1A,B) with the recovery rates of 80.9% and 86.4%, which indicated HNCP3 and UNCP4 might be homogeneous components polysaccharide^29^. Therefore, UAE was a more efficient technique to obtain higher polysaccharides yield from *N. commune* than HRE*,* and has more molecules with similar physical and chemical properties due to the cavitation, mechanical and heat effects synthetically^30^.

### Molecular weights of HNCP3 and UNCP4

The average molecular weights of HNCP3 and UNCP4 were evaluated by HPSEC-MALLS-RID technique as shown in Table 1 and Fig. 1C,D. All HNCP3 and UNCP4 samples exhibited the similar composition structures with different molecular weight distributions, indicating that HNCP3 and UNCP4 were heteropolysaccharides. The highest Mw of HNCP3 and UNCP4 were 48.6 kDa and 14.54 kDa, respectively, while the lowest Mn were 22.72 kDa and 44.32 kDa, respectively. As shown in Table 1, the averages molecular weight of UNCP4 was lower than that of HNCP3, and the low-molecular weight distribution of UNCP4 (68.57%) was greater than that of HNCP3 (46.18%), suggesting that the UAE method could produce a larger amount of the low Mw fractions than the traditional HRE method^31^. This might be attributed to the polysaccharides were degraded by ultrasonic to some extent, and a portion of the high-Mw fractions were converted into the low-Mw fractions, thereby increasing the amounts of the low Mw fractions. The same phenomenon was also observed in previous report^29^.

**Table 1:** Molecular weight distributions of HNCP3 and UNCP4 extracted by different methods.

Sample	Mw (Da)	Mn (Da)	Mw/Mn	%/area
HNCP3	4.86 × 10^4^	2.272 × 10^4^	2.443	46.18
UNCP4	1.454 × 10^4^	4.432 × 10^4^	3.281	68.57

*Mw* Weight-average molecular weight; *Mn* Number-average molecular weight; *Mw/Mn* Polydispersity index.

### Monosaccharide compositions analysis

**Figure 2:**
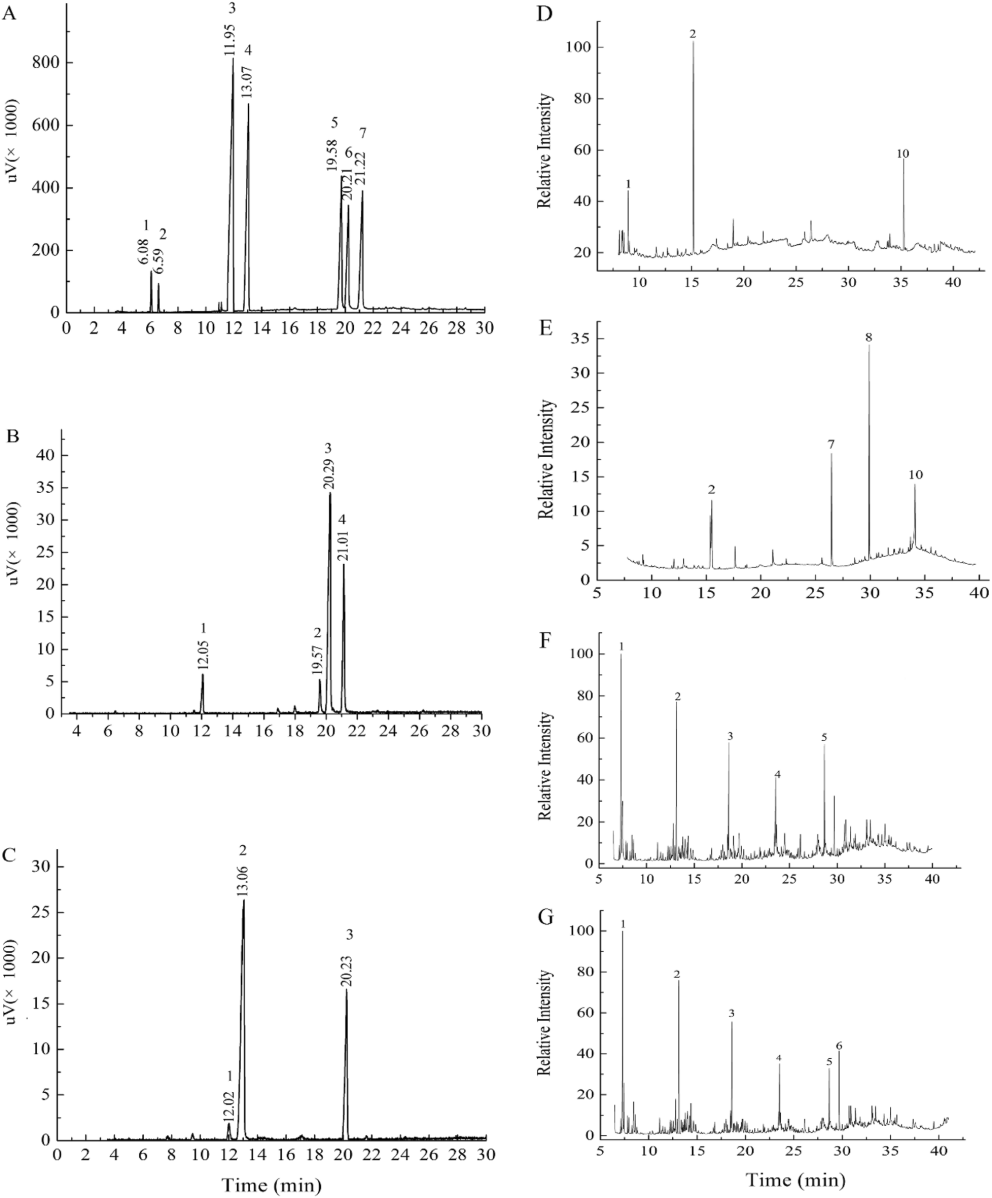
GC–MS spectra of the reference sample. (**A**) HNCP3; (**B**) UNCP4; (**C**) UNCP; (**D**) The total ion chromatogram from methylation analysis of HNCP by GC-MS; (**E**) The total ion chromatogram from methylation analysis of UNCP by GC-MS; (**F**) The total ion chromatogram of the methylated product of HNCP3; (**G**) The total ion chromatogram of the methylated product of UNCP4.

The monosaccharide compositions of samples were determined by GC–MS and the results were presented in Fig. 2. HNCP3 and UNCP4 were analyzed by matching their retention times with those of the standard monosaccharides (Fig. 2A), indicating the two polysaccharides had the different monosaccharide compositions. HNCP3 was composed of rhamnose, mannose, glucose, galactose in a molar ratio of 0.65: 0.55: 2.91: 2.73 and UNCP4 was composed of mannose, arabinose, glucose in a molar ratio of 0.30: 6.84: 2.81. The results illustrated that both HNCP3 and UNCP4 were enriched in glucose, indicating that glucose was the predominant monosaccharide. S. Jensen reported that the monosaccharide composition of Nc-5-s purified from an alkali extract of *N. commune* was Glc, GlcA, Xyl, Man, Ara, Rib, Gal in the ratio of 24: 24: 15: 13: 13: 7: 4. While the monosaccharide of a water soluble polysaccharide (NSKP) purified by precipitating hot-water extract from *Nostoc* with 40% (v/v) ethanol hot-water was composed of mannose, glucose, xylose, galactose, and glucuronic acid with a molar ratio of 1.00: 1.92: 0.97: 1.02: 0.91^32^. These results demonstrated that the difference monosaccharide composition was caused by the different processing methods and material sources^33^.

### Linkage analysis of HNCP3 and UNCP4

According to the results of periodate oxidation reaction, the consumptions of sodium periodate of HNCP3 and UNCP4 were 0.61 mol and 0.93 mol, and the total amounts of formic acid produced were calculated to be 0.43 mol and 0.65 mol. The molar ratios of periodate acid consumption to formic acid production were 1.42 and 1.43, respectively, indicating that HNCP3 and UNCP4 might contain glycosidic bonds of 1 → 2, 1 → 2, 6, 1 → 3, 1 → 3, 6, etc^34^. The GC of smith degradation of HNCP3 was presented in Fig. 2D. The detection of glycerol suggested that HNCP3 may contain 1→ , 1→ 2, 1 → 6 and 1 → 2,6 glycosidic bond. The detection of erythritol demonstrated that the bond type of the formation of erythritol existed, namely 1 → 4 and 1 → 4, 6-hexose. Meanwhile, the detection of rhamnose, mannose, glucose and galactose revealed that the polymers maybe linked by four kinds of monosaccharides with 1 → 3, 1 → 2, 3, 1 → 3, 4, 1 → 3, 6 and 1 → 2, 3, 4 glycosidic bonds. The GC of smith degradation of UNCP4 was presented in Fig. 2E, the erythritol formation indicated the possible presence of 1 → 4 and 1 → 4, 6 bonds hexoses. The detection of mannose and glucose and the increasing of glucose content comparing with the samples without periodate oxidation exhibited that most of UNCP4 were not oxidized by periodate, and the polymer mainly linked with 1 → 3 or 1 → 2,3 or 1 → 2, 4 or 1 → 3, 4 or 1 → 3, 6 or 1 → 2, 3, 6 or 1 → 2,4,6 or 1 → 3, 4, 6 glycosidic bonds. Methylation analysis was carried out to determine the inter monosaccharides linkages in HNCP3 and UNCP4 by GC/MS (Fig. 2F,G), and the linkage details were shown in Table 2. The results demonstrated that the methylated products of HNCP3 were total six liberations of 2, 4, 6-Me3-Glcp [peak 1: → 3)-Glcp-(1 →], 2, 3, 6-Me3-Glcp [peak 2: → 4)-Glcp-(1 →], 3, 4, 6-Me3-Galp [peak 3: → 2-Galp-(1 →], 2, 4-Me2-Galp [peak 4: → 2, 6)-Galp-(1 →], 2, 3, 4-Me3-Glcp [peak 5: → 6)-Glcp-(1 →], 2, 3, 4-Me3-Galp [peak 6: → 6)-Galp-(1 →] in a relative molar ratio of 1: 1.34: 1.28: 1.98: 3.32: 3.12 (Table 2). No rhamnose was detected due to the lower molar ratio in this polysaccharide. The following repeating unit of HNCP3 was deduced as below:

**Table 2:** Methylation analysis of HNCP3 and UNCP4 by GC–MS.

Polysaccharide	Rt	Methylated sugar	Linkage	Molar ration
HNCP3	8.09	2, 4, 6-Me3-Glcp	→ 3)-Glcp-(1→	10.56
	11.96	2, 3, 6-Me3-Glcp	→ 4)-Glcp-(1→	14.17
	13.16	3, 4, 6-Me3-Galp	→ 2-Galp-(1→	13.58
	18,76	2, 4-Me2-Galp	→ 2, 6)-Galp-(1→	21.01
	24.82	2, 3, 4-Me3-Glcp	→ 6)-Glcp-(1→	35.06
	29.23	2, 3, 4-Me3-Galp	→ 6)-Galp-(1→	32.93
UNCP4	9.38	2, 4-Me2-Rhap	→ 3)-Rhap-(1→	1.02
	14.76	2, 3-Met-Glcp	→ 4, 6)-Glcp-(1→	25.12
	20.93	2, 3, 4-Me3-Glcp	→ 6)-Glcp-(1→	36.85
	24.68	2, 3, 4, 6-Me4-Glcp	→ 4)-Glcp-(1→	42.15
	30.07	2, 3, 6-Men-Glcp	→ 3)-Glcp-(1→	10.07
	32.36	2, 4-Me2-Glcp	→ 3, 6)-Glcp-(1→	18.13


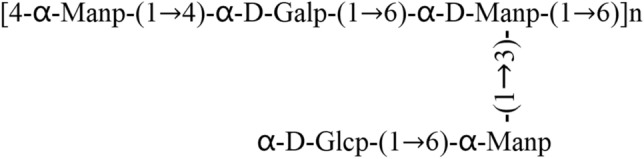


The methylated product of UNCP4 was composed of the 2, 4-Me2-Rhap [peak 1: → 3)-Rhap-(1→], 2, 3-Met-Glcp [peak 2: → 4, 6)-Glcp-(1 →], 2, 3, 4-Me3-Glcp [peak 3: → 6)-Glcp-(1 →], 2, 3, 4, 6-Me4-Glcp [peak 4: → 4)-Glcp-(1 →], , 3, 6-Men-Glcp [peak 5: → 3)-Glcp-(1 →], 2, 4-Me2-Glcp [peak 6: → 3, 6)-Glcp-(1 →] in a relative molar ratio of 1: 24.63: 36.13: 41.32: 9.87: 17.78 (Table 2). The results revealed that the presence of the following repeating unit in the UNCP4.
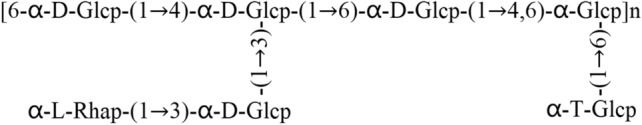


At present, a variety of plant polysaccharides have been reported to have antioxidant activities, including green algae, brown algae and red algae and other algal polysaccharides^35–37^. It has been reported that most polysaccharides with outstanding biological activities are linked by (1 → 3) glycosidic bonds, which have β–(1 → 3)-D-glucan backbone structure with some side chains^38,39^. However, the structure and stereo configuration of plant polysaccharides are very complex, and their structure–function relationship remains to be further studied. An edible mushroom (*Calocybe indica*) has been reported that possesses immunostimulatory activity and the primary structural backbone has the same α-D-Glcp-(1 → 4) glycoside unit as HNCP3 and UNCP4^40^. *Toona sinensis* leaves polysaccharide has good antioxidant and other protective effects against acute liver injury in mice^41^. Wherein, the *Toona sinensis* leaves polysaccharide TSP-1 main chain has the same α-D-Manp-(1 → 6) glycosidic unit as HNCP3 and the same α-D-Glcp-(1 → 6) glycosidic unit as UNCP4. While the branched chain has the same α-D-Glcp-(1 →) glycosidic unit as HNCP3 and the same α-D-Manp-(1 → 3) glycosidic unit as UNCP4. The detection of the primary structure of *Nostoc commune* polysaccharide proved that its polysaccharide main chain has α-D-Glcp-(1 → 4), α-D-Manp-(1 → 6) or α-D-Glcp-(1 → 6) glycoside, while the side chain has α-D-Glcp-(1 →) or α-D-Manp-(1 → 3) glycoside, so it was speculated to have potential antioxidant activity. In a word, these methylate results were consistent with the observations of periodate oxidation and smith degradation of HNCP3 and UNCP4.

### FT-IR spectrum and TGA/DSC detection

**Figure 3:**
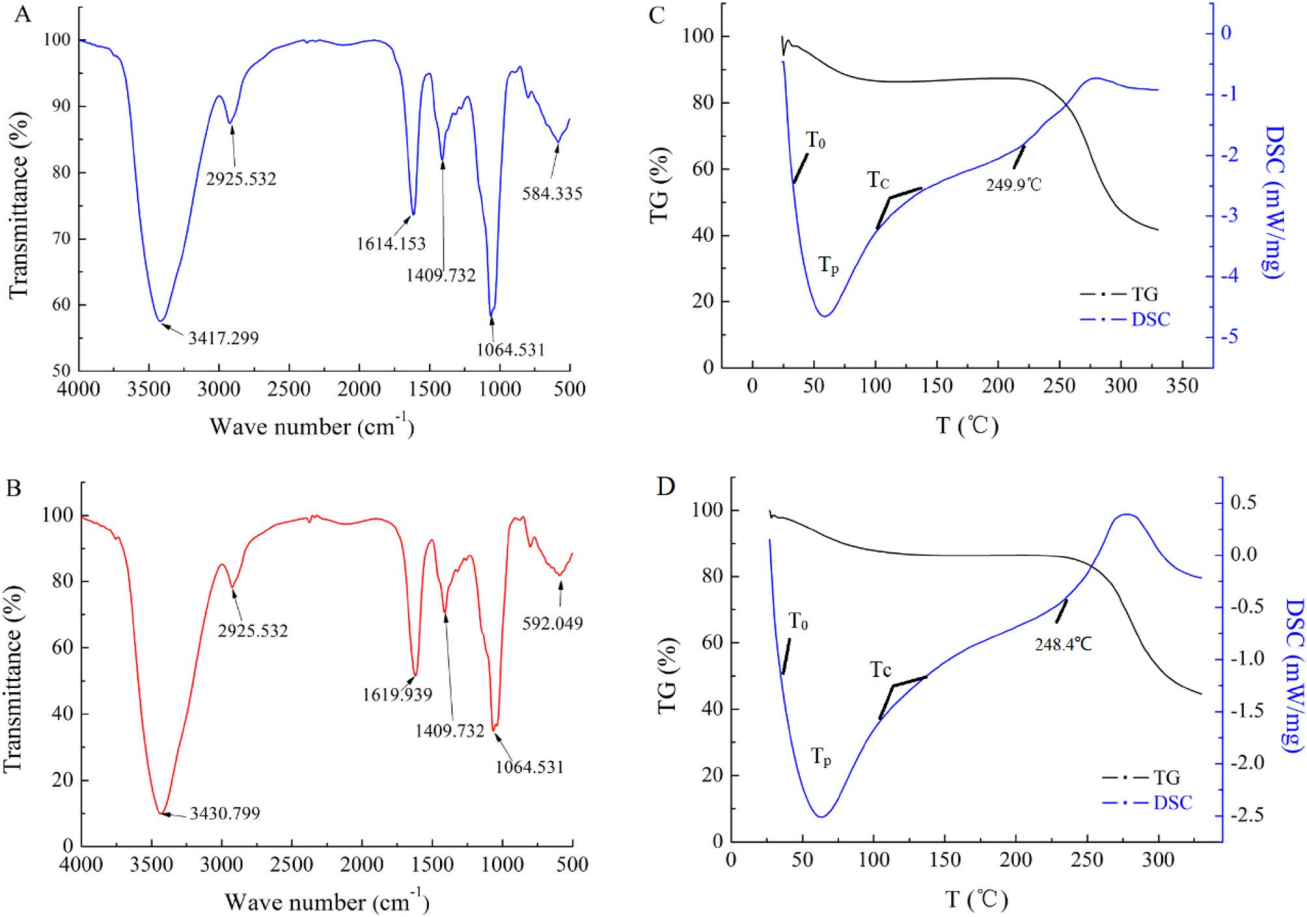
FT-IR spectra of HNCP3 (**A**) and UNCP4 (**B**). (NCP are *Nostoc commune* polysaccharides). DSC detects HNCP3 (**C**) and UNCP4 (**D**).

No visible differences could be observed between the spectra of the HNCP3 and UNCP4 as displayed in Fig. 3A,B, indicating that the HNCP3 and UNCP4 extracted by the different methods had similar functional groups. In details, the absorptions were very obvious at 3400 cm^−1^ and 1100 cm^−1^, that were caused by the stretching and angular vibration of O–H linkage^42^. The absorption at 3423.085 cm^−1^ was attributed to the C-H stretching vibration. An asymmetrical stretching peak at 1637 cm^−1^ and an absorption peak approximately at 1412 cm^−1^ were the indicators of the presence of carbonyl and carboxyl groups stretching vibration^43,44^. The two absorption peaks at 1064.531 cm^−1^ and 1062.603 cm^−1^ corresponded to the stretching vibrations of C–O–C or C–O–H of a pyranose ring, confirming that HNCP3 and UNCP4 contained pyranose sugar^45^. The mid-peak at 590 cm^−1^ and the weak peak at 586 cm^−1^ were associated with the presence of α and β-glycosidic linkages, respectively.

The thermogravimetric (TG) spectrum was used to determine the weight loss ofHNCP3 and UNCP4 on heating from 25 to 300 °C. As shown in Fig. 3C,D, the results revealed a one stage weight loss corresponding to the loss of water around 25–248 °C. The curve showed that UNCP4 did not decompose before 248 °C with a relatively smoothly weight loss of 10.97% at 71.97 °C, while HNCP4 began to decompose at 216 °C with a faster weight loss of 13.35% at 90.09 °C. It has been reported^46^ that water was formed by intra- and intermolecular condensation of polymer hydroxyls, and the decomposition of polymer occurred at temperatures below 300 °C. The weight loss rate of UNCP4 was lower than that of HNCP3, indicating that UNCP4 might has good thermal stability or has the rumbly textures and coarse unconsolidated surfaces^47^.

Differential scanning calorimetry (DSC) was applied to determine the exothermal or endothermal changes with the temperature increasing of HNCP3 and UNCP4 (Fig. 3C,D). In which, the three key temperature points involved in the energy change were marked as T_0_ (initial temperature), T_p_ (intermediate temperature)_,_ and T_c_ (final temperature). Both polysaccharides exhibited amorphous portions. The glass transition temperatures of HNCP3, UNCP4 occurred at 61.2 °C and 62.8 °C without melting peaks. The continuous (broad) endothermic transitions were observed as an indicative of moisture loss in HNCP3 and UNCP4. Figure 3C displayed the enthalpy change value of HNCP3 was − 211.6 J/g and the exothermic peak was relatively gentle with the temperature of exceeding 249.9 °C, and the pyrolysis reaction proceeded relatively smoothly. Nevertheless, the enthalpy change value of HNCP3 was − 226.1 J/g and a steep exothermic peak occurred at the temperature over 248.4 °C and the pyrolysis reaction proceeded more vigorously (Fig. 3D). In a word, there was a little difference between both polysaccharides by the DSC curves analysis.

### SEM and AFM observations of HNCP3 and UNCP4

**Figure 4:**
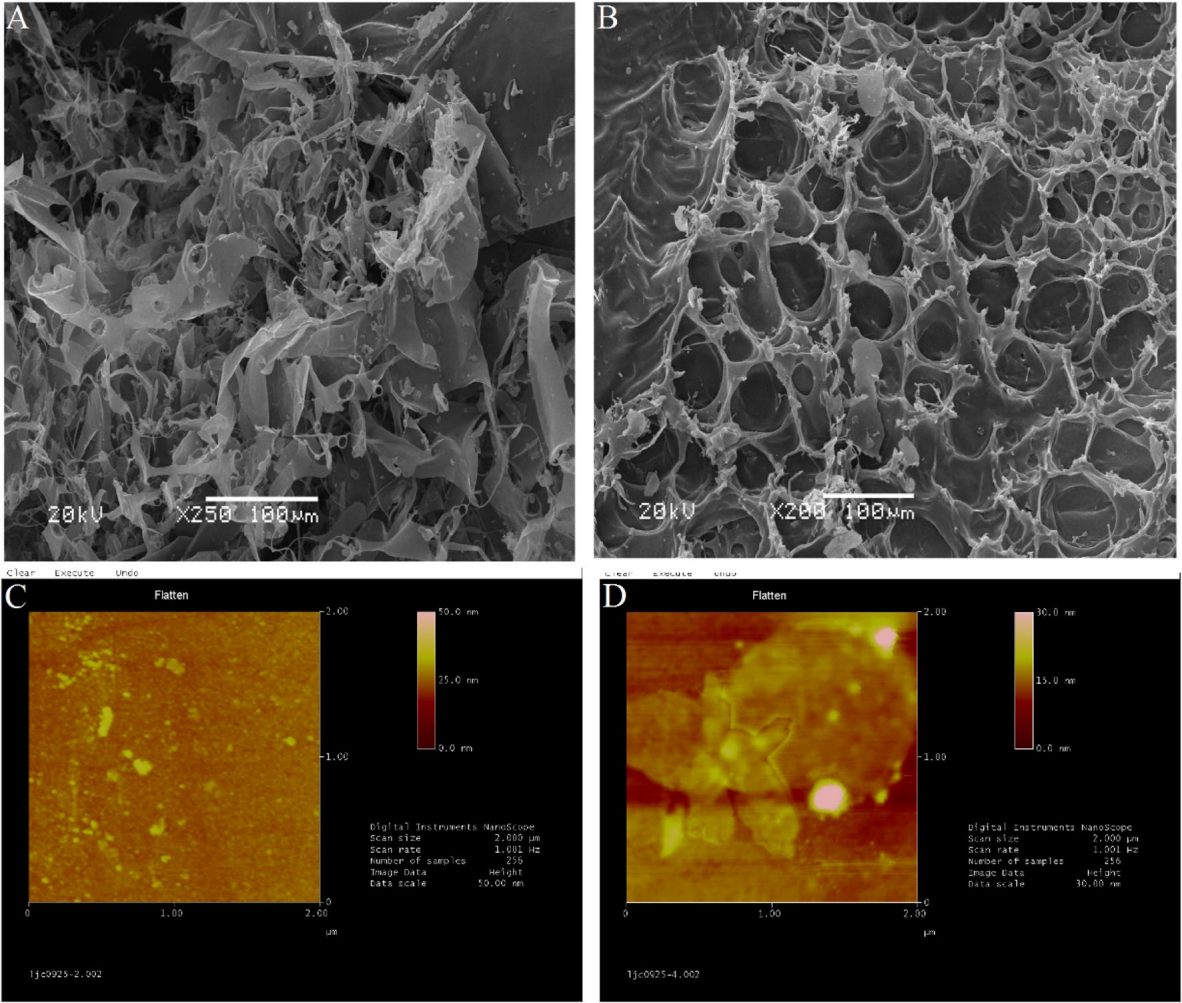
Scanning electron micrographs of *Nostoc commune*: SEM images of HNCP3 and UNCP4 obtained by JSM-7001E microscope in the scale bar of 100 p. m (**A**) and 10 p. m (**B**); (**C**, **D**) Atomic force micrographs of HNCP3 and UNCP4 obtained under tapping mode by using a Multimode 8 instrument (Bruker, USA).

Different extraction methods could affect the microstructures of polysaccharides^48,49^, the morphology and structures of HNCP3 and UNCP4 were observed by SEM and AFM (Fig. 4) in this paper. At the resolution of 100 μm, the surface of HNCP3 was smooth and irregularly jagged with a filamentous and sheet-like connection (Fig. 4A), and UNCP4 exhibited the higher smooth and uniform surface with the pronounced honeycomb-like and helical voids structure (Fig. 4B), which indicated that the changes between the two polysaccharides might be due to the long heating time, high temperature, and the cavitation effect caused by ultrasonic vibration. These results were similar to previously reported results that UAE had a more drastic effect on the microstructure of polysaccharides^50^.

To further observe the fine structures of HNCP3 and UNCP4, the AFM was used for the morphological observation. As shown in Fig. 4C, D, HNCP3 and UNCP4 molecules were ellipsoidal. UNCP4 molecules were loosely packed and uniform in size and shape, and the horizontal length of the molecules was 226.56 nm with the vertical height of 7.126 nm. Compared to UNCP4, HNCP3 molecules showed different sizes and shapes with large number of spherical micelles and a small amount of dispersion, and the horizontal length of the molecules was 101.56 nm with the vertical height of 13.961 nm. According to above results, we could speculate that molecular aggregation existed in UNCP4, and its structural units might branch and entangle with each other. The SEM and AFM analysis provided strong evidences for the improved efficiency of polysaccharide extraction by UAE.

### Antioxidant activities of HNCP3 and UNCP4

**Figure 5:**
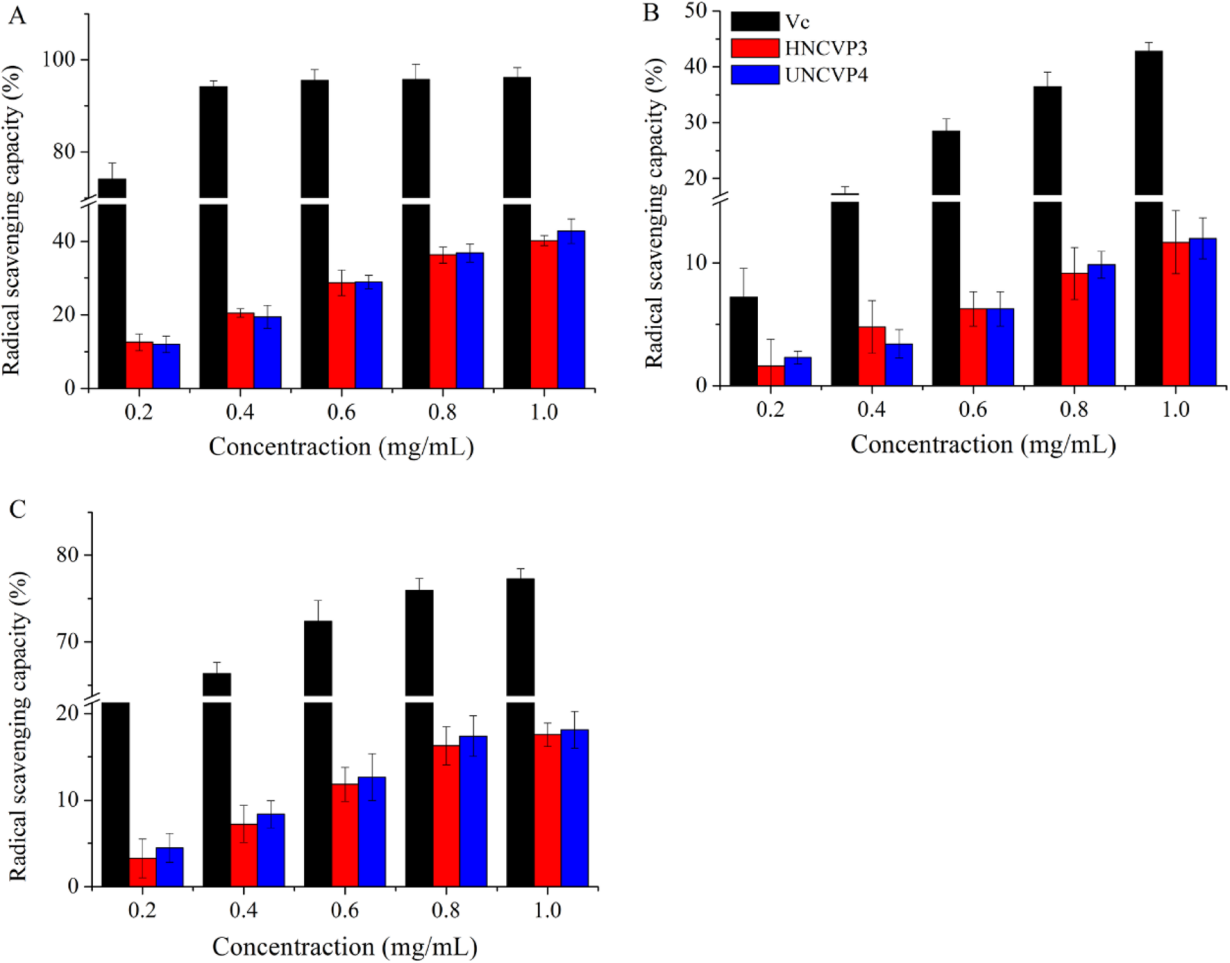
Antioxidant activities of HNCP3, UNCP4 and vitamin C (Vc). Note: (**A**) Effect of different concentration of samples on DPPH radical scavenging activities; (**B**) Effect of different concentration of samples on removal of hydroxyl radicals; (**C**) Effect of different concentration of samples on removal of superoxide anion.

Various mechanisms of anti-oxidation have been reported that including suppression of chain initiation, chelating metal ions, peroxides decomposition and radical scavenging^51,52^. However, the mechanism of action of the antioxidant activities of HNCP3 and UNCP4 remained unclear. The antioxidant activities of HNCP3 and UNCP4 were estimated and presented in Fig. 5, that were the free radical cleaning rates gradually increased with increasing concentrations of the samples. Figure 5A showed the scavenging activities of HNCP3 and UNCP4 on the DPPH· radical compared with Vc. Among all the samples, the positive control Vc solution had the highest clearance rate, reaching the maximum of 95.41% at the concentration of 0.2 mg/mL. UNCP4 also exhibited relatively stronger scavenging capacity (52.72%) than HNCP3 (40.21%) at the concentration of 1.0 mg/mL. The results of the removal of superoxide anion radicals by HNCP3 and UNCP4 were shown in Fig. 5B. The scavenging ability increased significantly with the rise of the concentration from 0.2 to 1.0 mg/mL, and the maximum clearance rates of HNCP3 and UNCP4 at 1.0 mg/mL were 30.12% and 26.02% respectively. Figure 5C exhibited the effects of HNCP3 and UNCP4 on scavenge ·OH, with the highest clearance rates reaching 22.71% and 26.70% respectively, that were significantly lower than the scavenging effect of Vc on hydroxyl radicals. Previous studies have reported that the scavenging effect of crude polysaccharide (HRSA) of *N. commune* on HO·could reach at 92.71% with the concentration of 10 mg/mL^53^. The mechanisms of the antioxidant action of HNCP3 and UNCP4 might be the molecule’s ability to supply hydrogen atoms to free radicals, thus terminating free radical chain reactions and converting free radicals to unharmful products^15,54^. In this paper, HNCP3 and UNCP4 showed different antioxidant activities, that were attributed to their monosaccharide composition ratios and side-chain linkages. In addition, ultrasonic wave might appropriately degrade the high-molecular-weight polysaccharides and change their antioxidant activities^55^.

It has been reported that physical properties such as molecular weight, solubility and viscosity of polysaccharides, as well as the extraction method of polysaccharides could affect the antioxidant effect of plant polysaccharides^56^. Yang et al. found that hot water extraction and ultrasound-assisted extraction of *Pleurotus citrinopileatus* polysaccharides had stronger free radical scavenging ability compared to alkali extraction^57^. Moreover, Ni et al. studied the monosaccharide composition of eight plant polysaccharides and their DPPH scavenging activity. The results showed that the DPPH radical scavenging ability of the eight polysaccharides was not related to the polysaccharide content but to the microstructure, and the specific way and intensity of the effect of monosaccharide composition on the antioxidant activity of the polysaccharides need to be further investigated^58^. In this study, when the concentration of polysaccharides extracted by two kinds of extract methods was 1 mg/mL, the antioxidant effect of UNCP4 was slightly stronger than that of HNCP3, but not significantly.

### Inhibition rates of α-amylase and α-glucosidase

**Figure 6:**
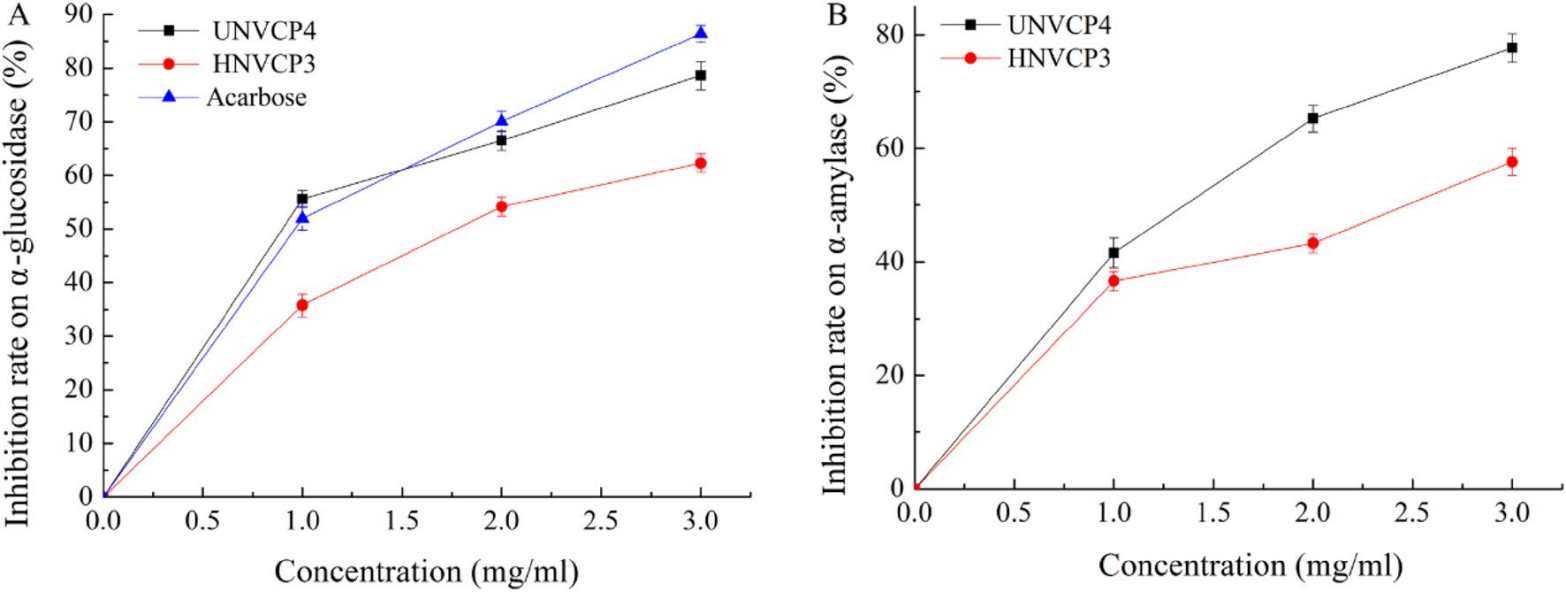
Inhibition rates of α-amylase and α-glucosidase by HNCP3 and UNCP4.

HNCP3 and UNCP4 could inhibit the activities of α-amylase and α-glucosidase, which were involved in glucose metabolism by reducing the cleavage of carbohydrate glycosidic bonds and releasing glucose. As shown in Fig. 6A, the inhibition rate of acarbose on α-glucosidase reached the maximum of 86.42 ± 1.59% when the concentration of samples was 3.0 mg/ml, and the highest inhibition rates of HNCP3 and UNCP4 were also obtained with the value of 60.03 ± 1.58% and 79.01 ± 1.41%, respectively. Meanwhile, the inhibitory activities of HNCP3 and UNCP4 on α-glucosidase were showed in Fig. 6B, where the inhibition ratio presented the dose-dependent relationship. The highest inhibition rates of HNCP3 and UNCP4 on α-amylase were 57.76 ± 1.88% and 77.72 ± 2.03%, respectively. The results showed that HNCP3 and UNCP4 could prevent the metabolism of carbohydrates in food and effectively reduce postprandial blood glucose levels in the body^59^.

In fact, it has been found that many extracts from plants and fruits possessed inhibitory effects on α-glucosidase with minimal side effects^60^. Although HNCP3 and UNCP4 had lower inhibitory effects than the drug acarbose, the results indicated they could be the potential inhibitors of α-glucosidase. In this study, the difference in the inhibitory activities of HNCP3 and UNCP4 might be due to the fact that ultrasound-assisted extraction could extract and/or preserved more bioactive compounds with anti-hyperglycemia activity in a short time under medium temperature conditions^61^.

*Nostoc commune* is rich in protein, calcium, phosphorus, iron and other nutrients, and has been used as an ingredient for a long time for human consumption, with the effect of lowering fat, clearing heat and brightening eyes, etc. The biological activity of polysaccharide, the important active ingredient in *Nostoc commune*, is not clear. In recent years, with the vigorous development of antioxidants, it has gradually developed from simple synthetic antioxidant to natural, green, efficient, low-toxicity free radical scavenger in vivo obtained from natural products. Diabetes is one of the three most persistent diseases threatening human health, with a mortality rate second only to cardiovascular diseases and cancer. The development of therapeutic drugs for diabetes has been a hot research topic. Many types of polysaccharides have been found to have certain antidiabetic activity in natural product components. Therefore, in this study, *Nostoc commune* polysaccharides extracted by hot water extraction and ultrasonic-assisted extraction were purified and structurally characterized. Their antioxidant and anti-diabetic activities were also investigated exploratively. The results showed that HNCP3 and UNCP4 have certain activities of scavenging free radicals.

α-amylase and α-glucosidase are important enzymes in the digestion of carbohydrates in food. α-amylase inhibitors and α-glucosidase inhibitors can inhibit the activity of α-amylase and α-glucosidase, slow down the conversion and absorption of glucose, reduce the postprandial blood glucose peak, and adjust the blood glucose level, thus reducing the stimulation of blood glucose to the pancreas, improving the sensitivity of insulin, protecting the function of the pancreas, and effectively preventing and improving the occurrence and development of diabetes. In this study, the in vitro inhibitory activities of polysaccharides extracted by both methods against α-amylase and α-glucosidase were examined, and it was clear that both HNCP3 and UNCP4 have certain antidiabetic effects.

## Conclusions

In the study, the average molecular weight of HNCP3 was greater than the average molecular weight of UNCP4. Infrared spectroscopy indicated the conformational characteristics of UNCP4 and HNCP3, that is, the two polysaccharides had similar characteristic absorption peaks (O–H stretching vibration, C–H stretching vibration, C–H variable angular vibration and C–O bond) and different peak transmittances. AFM and SEM images showed molecular aggregation and polysaccharide conformation. HNCP3 was loose and presented a soft fibrous texture, while UNCP4 was dry and had a few branches on the surface. The monosaccharide component assay showed that the monosaccharide composition of HNCP3 and UNCP4 differed only in the molar ratio. The comparison of antioxidant activities of the two polysaccharides indicated that UNCP4 had strong antioxidant and hypoglycemic activities. In summary, Ultrasonic-assisted extraction had a certain effect on the structure and solution conformation of NCP, especially the effect on solution properties and chain conformation could affect the biological activity of polysaccharides. Therefore, it is of great significance to systematically study the effect of ultrasound on the advanced structure of traditional Chinese medicine polysaccharides.

## Materials and methods

### Materials

*Nostoc commune* is a plant belonging to the genus *Nostocaceae*, *Cyanobacteria*, *Nostocaceae*. Morphologically, it is initially gelatinous and spherical, and later expands into lamellae, up to 10 cm, like a gelatinous cortex, dark olive or tea-brown, dark brown or black after drying. The algal filaments are curled, and only at the perimeter of the group there are obvious gelatinous sheaths, yellowish brown, thick and laminar, and constricted at the transverse septum. The air-dried fruiting bodies of *N. commune* were obtained from Gansu Kangxinyuan Ecological Agriculture Technology Development Co., Ltd (Lanzhou, Gansu Province, China. The production of raw materials shall refer to the food safety enterprise standard Q/SXZN0001S-2019 Ground Soft. Plants (either cultivated or wild), including the collection of plant material, complied with relevant institutional, national, and international guidelines and legislation.), powdered into fine powder of 0.178 mm by a high-speed pulverizer (FZ102, Beijing Zhongxing Weiye Instrument Co., Ltd., Beijing, China), and stored at room temperature until used. Monosaccharide standards (i.e., rhamnose, arabinose, galactose, glucose, xylose, mannose, fucose, glucuronic acid) α-amylase were purchased from Solarbio Science & Technology Co., Ltd. (Beijing, China), 1,1-diphenyl-2-picrylhydrazyl (DPPH), Acarbose solution, p-nitrophenol glucopyranoside (PNPG) and α-glucosidase were purchased from Sigma-Aldrich (USA). All other reagents were of analytical grade.

### Heated reflux extraction

HRE was performed based on the previously reported method with some modifications^62^. The powder of *N. commune* (5 g) was reflux extracted with deionized water (250 mL) in a flat-bottom flask at 85 °C for 190 min, and the extract was treated three times by Savage method (Chloroform: butyl alcohol in 4: 1 ratio) to remove protein. The deproteinizing solution was precipitated (4 °C for 12 h) by the addition of anhydrous ethanol to a final concentration of 80% (V/V), and the precipitate was lyophilized by vacuum freeze dryer (SCIENIZ-18 N, Ningbo Biotechnology Co., Ltd., Ningbo, China) to obtain crude polysaccharide of *N. commune* (HNCP).

### Ultrasonic-assisted extraction

The detailed UAE procedure of crude polysaccharides (UNCP) was carried out as our described previously^63^. UNCP was extracted under the optimal extraction condition at solid–liquid ratio of 1: 50, solvent of anhydrous ethanol, extraction temperature of 353.15 K, ultrasonic power of 540 W, extraction time of 25 min, and then were treated according to the description in “Monosaccharide compositions analysis”section.

### Purification of crude polysaccharide and determination of average molecular weights

200 mg of the HNCP and UNCP were prepared into a 10 mg/mL solution, and loaded into DEAE-52 anion-exchange chromatography column (2.6 × 45 cm), respectively, and eluted with 0–1.0 mol/L stepwise NaCl gradient at the flow rate of 1 mL/min. The HNCP3 and UNCP4 fractions were collected with 0.3 mol/L NaCl solution, dialysed (MWCO: 8–14 kDa) and lyophilized with the yield of 24.5 mg. The freeze-dried sample were further purified by Sephadex G100 with the deionized water at the flow rate of 1 mL/min for further study. The molecular weight of HNCP3 and UNCP4 were determined by high-performance liquid chromatography coupled with multi-angle laser light scattering as we previously described.

### Modification of chemical and physical properties of periodate-oxidized polysaccharides

As described in previous report, the monosaccharide compositions of HNCP3 and UNCP4 were achieved by gas chromatography (GC)^63^, the detailed procedure was according to the reported methods with some modification. 30 mg of HNCP3 and UNCP4 was mixed with the 30 mL of sodium periodate solution (15 mmol/L), and the mixtures were sufficiently dissolved with a magnetic stirrer in a dark room at 4 °C. 1.0 mL of mixed solution was pipetted into the ampoule, placed into 250 mL volumetric flask and diluted to volume with deionized water. The absorbance of the solution at 223 nm was measured by ultraviolet spectrophotometer at intervals of 6 h until the absorbance value remained constant. The consumption of periodate was calculated with the sodium periodate calibration. 2.0 mL of the reaction solution was added 0.5 mL of ethylene glycol to terminate the reaction and was titrated by NaOH standard solution (0.01 mol/L) with the indicator of phenolphthalein. The volumes of titrant were recorded and the amount of formic acid was calculated according to the following formula 1.

1 $${\text{y}} = (x \times {n \mathord{\left/ {\vphantom {n 2}} \right. \kern-0pt} 2}) \times v$$

Note y is formic acid production, x is accurate concentration of NaOH, n is volume for titration, v is reaction volume.

The GC chromatographic conditions were as follows: ThermoITQ1100 Gas Chromatograph; FID detector; HP-5 flexible quartz capillary column (0.32 mm × 0.25 μm × 30 m); N_2_ as carrier gas; 1 mL/min as flow rate; 1: 10 as split ratio; 220 °C as inlet temperature; 250 °C as detector temperature; programmed ramp-up (160 °C as the starting temperature and ramped up to 240 °C at 10 °C/min).

The HPLC chromatographic conditions were as follows: the column was TSK-GELG6000PWXL; the mobile phase was 0.2% sodium iterate aqueous solution, the flow rate was 1 mL/min, the column temperature was 30 °C, the detector was a differential refractive index detector, and the injection volume was 20 μL.

### Smith degradation and methylation analysis of HNCP3 and UNCP4

The above periodic acid oxidation reaction solution terminated by ethylene glycol was transferred to a dialysis bag (3500 Da) and dialyzed with the distilled water for 48 h. 100 mg of sodium borohydride was added and mixed in the dark for 24 h, then the solution was further neutralized with 50% acetic acid until the excess sodium borohydride was decomposed. The neutralized solution was dialyzed for 48 h, and dried by freeze drying. The dried sample (10 mg) was hydrolyzed with 2 mL of trifluoroacetic acid (2 mol/L) at 120 °C for 3 h, and repetitive evaporated with methanol to remove the excess trifluoroacetic acid. The residue was treated with the mixture of 0.5 mL of pyridine and 10 mg of hydroxylamine hydrochloride at 95 °C for 30 min, and then acetylated with 0.5 mL of acetic anhydride at 95 °C for 35 min. The acetylate was blow-dried with nitrogen, dissolve with 1 mL of chloroform, washed twice with distilled water. 1 mL of chloroform layer solution was used to GC determination under the analytical conditions we establised^64^. 5 mg of the monosaccharide standard, glycerin and erythritol were taken respectively and performed GC analysis according to the method described above.

### Methylations of HNCP3 and UNCP4 were conducted to analyze the linkage patterns of polysaccharide

In detail, 10 mg of HNCP3 and UNCP4 were dissolved in 1.5 mL of DMSO (1.5 mL), then 1.5 mL of NaOH (50%)-DMSO solution (V:V = 1:1) was added and reacted at 30 °C for 3 h. Methyliodide (0.5 mL) was added and stirred at 30 °C for 2.5 h in dark place under N_2_ protection, and the reaction was terminated with 1 mL of deionized water. The above operation step was repeated three times until the absorption peak of OH group disappeared substantially. The methylated HNCP3 and UNCP4 were further hydrolyzed with H_2_SO_4_ (2 mol/L) at 120 °C for 2 h and dried by rotary evaporation under the protection of nitrogen. The rotary-dried sample was dissolved with NaOH solution, and shaken with the adding of 25 mg of sodium borohydride at 25 °C for 2 h, the pH was adjusted to 5.5–7.0 with acetic acid for removing excess sodium borohydride.

Subsequently, the reduced samples were acetylated by mixing with 0.7 mL of pyridine and 1 mL of acetic anhydride at 90 °C for 30 min. The dried reaction product was dissolved in ethyl acetate and then analyzed by a GLC-MS system (THERMO 1310 GC-ISQ LT MS, USA) apparatus equipped with a TG-200MS capillary column (30 mm × 0.25 mm, 0.25 µm). The specific program was as follows: from 160 to 210 °C, the temperature was raised at a rate of 2 °C/min, then increased to 240 °C at a rate of 5 °C/min, and finally kept for 20 min. MS scan range was set at m/z 35–4000.

### FT-IR spectroscopy analysis and TGA/DSC measurement

FT-IR spectrophotometer (Nicolet iS5, Thermo Fisher Scientific, USA) was used to determine the organic functional groups of HNCP3 and UNCP4 in the scanning range of 4000–400 cm^−1^ with a resolution of 4 cm^−1^^65^. HNCP3 and UNCP4 were placed in Al_2_O_3_ crucible, and the temperature was raised to 350 °C at a rate of 10 °C /min with the shielding gas of nitrogen on TGA/DSC simultaneous thermal analyzer (STA449C, Netzsch, Germany)^66^. The denaturation temperature was calculated using the Origin 9.0 data analysis software.

### Molecular surface morphology observation of HNCP3 and UNCP4

In order to investigate the effect of different extraction methods on the microstructure of polysaccharides, HNCP3 and UNCP4 were fixed on gold-coated silicon wafers for and observed by scanning electron microscope (SEM, JSM-5600LV, American Kevex Company, America). Meanwhile, 50 µL of HNCP3 and UNCP4 (1.0 µg/mL) were dropped to mica substrate and dried at room temperature overnight. Then the conformational transformations of HNCP3 and UNCP4 macromolecules were studied by AFM (MultiMode-HR, Brooke, USA)^67^.

### In vitro antioxidant activities determination

According to our previously described method^65,68,69^, HNCP3, UNCP4 and Vc solutions with different concentrations (0.2 mg/mL, 0.4 mg/mL, 0.6 mg/mL, 0.8 mg/mL, 1.0 mg/mL) were prepared to evaluate their antioxidant activities in terms of DPPH radical scavenging ability, superoxide anion scavenging ability and hydroxyl radical scavenging ability. To be specific, 1 mL of crude polysaccharides of HNCP3 and UNCP4 with concentrations of 0.2 mg/mL, 0.4 mg/mL, 0.6 mg/mL, 0.8 mg/mL and 1.0 mg/mL were added into the test tubes. 5 mL of 5 mmol/L DPPH solution was then added, shaken well, and placed in the dark for 30 min. The mixture of 5 mL absolute ethanol and 3 mL distilled water was used as the reference, and the absorbance value at 517 nm was measured. The DPPH scavenging activity was calculated by formula 2.

2 $${\text{Scavenging activity }}\left( \% \right) \, = \, [A_{0} - (A_{i} - A_{j} )]/A_{0} \times \, 100\%$$

Note *A*_0_ is the absorbance of the blank control solution with distilled water instead of sample solution; *A*_*i*_ is absorbance of sample solution; *A*_*j*_ is Absorbance of sample solution with distilled water instead of chromogenic agent.

1 mL of crude polysaccharides of HNCP3 and UNCP4 with concentrations of 0.2 mg/mL, 0.4 mg/mL, 0.6 mg/mL, 0.8 mg/mL, 1.0 mg/mL and 3 mL of Tris–HCl buffer with a pH of 8.2 were added to the test tubes. The reaction was carried out in a water bath at 25 °C for 20 min. After adding 0.3 mL of 7 mmol/L pyrogallol and reacting for 4 min, 1 mL of 10 mol/L HCl was added, and the absorbance was measured at 420 nm. The superoxide anion scavenging ability was calculated by formula 2.

2 mL of crude polysaccharides of HNCP3 and UNCP4 with concentrations of 0.2 mg/mL, 0.4 mg/mL, 0.6 mg/mL, 0.8 mg/mL, 1.0 mg/mL, 1 mL of 9 mmol/L FeSO_4_ and 2 mL of 9 mmol/L salicylic acid–ethanol solution were added to the test tubes. Then 2 mL of 8.8 mmol/L H_2_O_2_ was added and reacted at room temperature for 1 h. The absorbance value of the samples was measured at 510 nm by zeroing with distilled water. The hydroxyl radical scavenging ability was calculated by formula 2.

### In vitro antidiabetic activity

The in vitro antidiabetic ability of HNCP3, UNCP4 was assessed by their inhibitory potential against α-amylase and α-glucosidase that could metabolize carbohydrate^67^. The sample concentration (IC_50_) for the ability to inhibit the enzyme by 50% was also calculated. Specifically, HNCP3 and UNCP4 were dissolved in phosphate buffer (0.2 mo1/L, pH 6.6) to concentrations of 1 mg/mL, 2 mg/mL, 3 mg/mL, 4 mg/mL, 5 mg/mL, 6 mg/mL, respectively. 0.2 mL of α-amylase solution (6 U/mL) was added into the sample solutions of different concentrations, followed by 0.4 mL of starch solution (1%), and the reaction was performed at 37 °C for 10 min. 2 mL of DNS reagents were sequentially added and reacted in boiling water batch for 10 min, the absorbance was measured at 520 nm as described in earlier reports^70^. The inhibition rate of α-amylase was calculated according to Formula 3.

3 $$\alpha {\text{ - amylase inhibition rate (\% )}} = \frac{{A_{0} - (A_{1} - A_{2} )}}{{A_{0} }} \times 100$$

Note *A*_0_ is the blank control absorbance, *A*_1_ is the absorbance of the polysaccharide sample, and *A*_2_ is the absorbance of the base tube.

The α-glucosidase inhibitory activity was determined by measuring the release of p-nitrophenol at 405 nm in a 96-well plate. The reaction mixture containing 20 µl of different concentrations of samples, 10 µl of α-glucosidase (2 U/ml), 50 µl of phosphate buffer (pH 6.8, 100 mM) was incubated at 37 °C for 15 min. Then, 20 μl of p-nitrophenol glucopyranoside (PNPG) (5 mM) was added and incubated at the same temperature for 20 min. The reaction was stopped by adding of 150 μl of sodium carbonate (0.1 M). Acarbose was used as standard. The inhibitory rate was calculated according to formula 4.

4 $$\alpha {\text{ - glucosidase inhibition rate }}(\% ) = \left( {1 - \frac{{A_{1} - A_{2} }}{{A_{3} - A_{0} }}} \right) \times 100$$

*A*_0_ is the blank absorbance, *A*_1_ is the absorbance of the polysaccharide sample, *A*_2_ is the blank absorbance of the sample, and *A*_3_ is the absorbance of the control.

### Statistical analyses

The data were expressed as mean ± SD and examined for their statistical significance of difference with variance (ANOVA) and T tests (and nonparametric tests) by GraphPadPrism5. P values less than 0.05 were considered to be statistically significant.

## Data Availability

The original contributions presented in the study are included in the article and Supplementary Material, further inquiries can be directed to the corresponding author.

## Abbreviations

ANOVA: Analysis of variance
NCP: *Nostoc commune* polysaccharide
HRE: Heating reflux extraction
MAE: Microwave-assisted extraction
UAE: Ultrasonic-assisted extraction
TG: Thermogravimetric spectrum
DSC: Differential scanning calorimetry
Vc: Vitamin C
HNCP3: *Nostoc commune* polysaccharide 3 obtained by heated reflux extraction
UNCP4: *Nostoc commune* polysaccharide 4 obtained by ultrasonic-assisted extraction
DPPH: 1,1-Diphenyl-2-picrylhydrazyl
PNPG: P-nitrophenol glucopyranoside
GC: Gas chromatography
FT-IR: Fourier transform infrared spectroscopy
IC_50_: Half maximal inhibitory concentration
DMSO: Dimethyl sulfoxide
GC-MS: Gas liquid chromatography mass spectrometry
TGA: Thermogravimetric analyzer
MWCO: Molecular weight cut-off
DEAE-52: Diethylaminoethyl-52
SEM: Scanning electron microscope
AFM: Atomic force microscope
HRSA: Hydroxyl radicals scavenging activity
